# Voluntary Exercise-Induced Skeletal Muscle Responses in Young and Aged Mice on a High-Fat Diet

**DOI:** 10.7759/cureus.86697

**Published:** 2025-06-24

**Authors:** Yuji Kanazawa, Kenichiro Miyahara, Tatsuo Takahashi, Ryo Miyachi, Takashi Higuchi, Takaaki Nishimura, Hiaki Sato, Yuri Ikeda-Matsuo

**Affiliations:** 1 Department of Physical Therapy, Hokuriku University, Kanazawa, JPN; 2 Department of Clinical Pharmacology, Hokuriku University, Kanazawa, JPN; 3 Department of Rehabilitation, Seijoh University, Nagoya, JPN; 4 Department of Medical Technology and Clinical Engineering, Hokuriku University, Kanazawa, JPN

**Keywords:** aging, high-fat diet, intramuscular fat, mitochondrial metabolic activity, voluntary exercise

## Abstract

Background and objective

Aging and consumption of a high-fat diet (HFD) are associated with increased body weight and reduced skeletal muscle quality. Although aerobic exercise is generally considered protective against these risks, the impact of self-initiated physical activity on an HFD in older individuals remains unclear. This study aimed to investigate the influence of spontaneous wheel-running on body weight, intramuscular fat accumulation, and mitochondrial metabolic function in the skeletal muscles of young and aged mice on HFD.

Methods

Male C57BL/6J mice aged 14 weeks (young group) and 84 weeks (aged group) were assigned to either exercise or sedentary groups and fed an HFD for eight weeks. Measurements included body weight, muscle weight (gastrocnemius and soleus), muscle fiber cross-sectional area (FCSA), succinate dehydrogenase (SDH) activity, and intramuscular fat area via Oil Red O staining.

Results

Voluntary exercise significantly reduced the body weight in both age groups. While the muscle weight and FCSA remained unchanged by exercise, exercise led to elevated SDH activity in the gastrocnemius muscles of both young and aged mice, suggesting increased mitochondrial metabolic activity. Exercise increased intramuscular fat content in the gastrocnemius muscle of young mice, but not in aged mice. The soleus muscle showed a minimal response to both metabolic activity and fat accumulation by exercise, regardless of age.

Conclusions

Voluntary wheel running under HFD conditions effectively lowered body weight and increased mitochondrial activity in gastrocnemius muscle fibers. However, intramuscular fat responses vary according to muscle type and age, suggesting that aging diminishes skeletal muscle adaptability to exercise in the context of lipid metabolism.

## Introduction

Advanced age is often associated with weight gain and a reduction in both skeletal muscle volume and functional capacity, factors that significantly contribute to the risk of metabolic diseases [1]. Additionally, intramuscular fat accumulation and reduced skeletal muscle metabolic function are important contributors to the frailty and decline in physical performance observed in older adults [2]. Obesity, often resulting from high-fat diet (HFD) consumption, exacerbates these issues by inducing contractile and metabolic dysfunction within the skeletal muscle tissue and increasing the risk of insulin resistance and related metabolic disorders [3,4]. Intramuscular fat accumulation associated with obesity has been reported to impair anabolic signaling by decreasing the activation (via phosphorylation) of molecular targets involved in the mTOR and AMPK signaling cascades, potentially leading to downstream consequences, including an impaired anabolic response and heightened susceptibility to sarcopenia [5-9]. These findings suggest that both aging and obesity contribute to the decline in skeletal muscle quality and quantity.

Exercise, particularly aerobic exercise, is a well-established intervention for managing body weight and mitigating the risk of metabolic diseases. Exercise promotes heat production and increases energy consumption through mechanisms involving the secretion of adipocytokines and myokines, anti-inflammatory responses, and antioxidant activity [10]. In older adults, exercise has been reported to increase the production of contractile proteins and promote capillarization in the skeletal muscles [11]. Previous studies using young rats or mice have demonstrated that voluntary exercise increases skeletal muscle oxidative capacity and upregulates the expression of myokines [12-15]. Increased skeletal muscle contractile function or metabolic activity has been observed in mice in the early stages of aging and late middle-aged rats subjected to voluntary exercise [16,17]. However, the effect of exercise intervention during an HFD feeding in aged mice, specifically on muscle metabolic activity and intramuscular fat accumulation, remains inadequately understood.

This study investigated the impact of unforced physical activity on lipid accumulation within skeletal muscle and the associated metabolic functions in both young and aged mice maintained on an HFD. Specifically, we investigated the effects of self-initiated exercise on body and skeletal muscle mass, mitochondrial metabolic activity, and intramuscular fat accumulation. By comparing the responses of young and aged mice, this study aimed to elucidate how aging affects exercise-driven adaptations related to muscle energy regulation and intramuscular fat storage, with a particular focus on the differences between the gastrocnemius and soleus muscles.

## Materials and methods

Animals, diet, and wheel running

This study involved 14-week-old (young; n = 10) and 84-week-old (aged; n = 8) male C57BL/6J mice (Jackson Laboratory, Kanagawa, Japan). Following delivery, all the mice underwent an acclimatization period of at least one week before the start of the eight-week experimental protocol. Within each age category, the animals were randomly distributed into groups and subjected to either voluntary wheel-running or sedentary conditions. All animals were fed an HFD (HFD-60, Oriental Yeast, Tokyo, Japan) throughout the study. The mice assigned to the exercise condition were individually housed in cages with access to a voluntary running wheel (Model ACT-557-WLP; Actimetrics, IL; cage dimensions: W270 × L440 × H187 mm). To ensure uniform housing conditions, sedentary mice were placed in single-occupancy cages that matched the configuration of the exercise cages, excluding access to the wheel. Throughout the study, animals were maintained under stable housing conditions (22 ± 2 °C, 12-hour light/dark cycle) with ad libitum access to food and water.

During the eight-week intervention period, voluntary exercise had no significant effect on weekly food intake in either age group, and there was no significant difference in the amount of voluntary exercise per week between the two age groups. The experimental protocol was approved by the Hokuriku University Animal Welfare Committee (approval number: 24-13; March 15, 2024) and complied with the university’s established regulations for laboratory animal care and use. In the present study, a power analysis was performed using G*Power 3.1 based on an effect size of f = 0.4, α = 0.05, and power (1−β) = 0.80, resulting in a total sample size of 52. Furthermore, since this study is an animal experiment, we considered reducing the number of animals used based on the 3R principles (replacement, reduction, refinement) and taking into account the limited number of aged mice available. Therefore, this study was conducted using a total of 18 mice: five young mice (no exercise), five young mice (exercise), four aged mice (no exercise), and four aged mice (exercise).

Sampling

All mice were weighed and euthanized subsequently via cervical dislocation, and the gastrocnemius and soleus muscles were excised and weighed. These muscles were frozen in isopentane pre-cooled with dry ice and stored at −80 °C for subsequent analysis.

Histochemical analysis

Transverse sections 10 μm thick were prepared from the belly of the lateral head of the gastrocnemius and the soleus muscle using a cryostat (CM1950; Leica, Wetzlar, Germany) at -25 °C. Sections were affixed to glass slides and stained with hematoxylin and eosin (HE). To visualize the nuclei, the slides were immersed in hematoxylin solution for 10 minutes, followed by eosin for one minute. In succinate dehydrogenase (SDH) staining, the incubation medium was composed of 0.2 M phosphate buffer (pH 7.6), 0.2 M sodium succinate, and 0.05% nitroblue tetrazolium. The sections were stained in the incubation medium at 37 °C for 45 minutes. After staining, all sections (HE and SDH) were dehydrated using sequential ethanol dehydration steps, cleared in xylene, and sealed using a mounting medium (Falma Inc., Tokyo, Japan). Sections were stained with Oil Red O to evaluate lipid droplet accumulation within the muscle fibers. Slides were pre-incubated in propylene glycol, incubated in Oil Red O stain (ORK-1, ScyTek Laboratories, Inc., UT) at 50 °C for 40 minutes, and rinsed with 85% propylene glycol. The nuclei were counterstained with hematoxylin, and the slides were promptly encapsulated using a water-soluble encapsulant (Fluoromount; Diagnostic BioSystems Inc., CA).

Morphological analysis

The muscle fiber cross-sectional area (FCSA) was quantified from the HE-stained sections using the Hybrid Cell Count and Macrocell Count tools within the BZ-X810 microscope analysis software (Keyence, Osaka, Japan), following the manufacturer’s recommended procedures. At least 450 fibers per gastrocnemius and over 100 fibers per soleus sample were analyzed. SDH staining intensity, used as an index of SDH activity, was measured from six images (393,880 μm^2^ per image) per gastrocnemius sample and one image per soleus sample. The intensity values were normalized by setting the mean value of the young sedentary group to 100 for each muscle, and comparisons were made across the groups. Oil Red O-stained areas were quantified from six images (393,880 μm^2^ per image) per gastrocnemius sample and one image per soleus sample. All image analyses were conducted by an evaluator blinded to the group assignments using the same software.

Statistical analysis

Statistical analyses were performed using EZR (Saitama Medical Center, Jichi Medical University, Saitama, Japan) [18], a graphical user interface for R (R Foundation for Statistical Computing, Vienna, Austria). This software is a modified version of R Commander, tailored to incorporate functions commonly used in biostatistics. A two-way analysis of variance was used to evaluate the main effects of age, exercise, and their interactions. When a significant interaction was detected, post hoc comparisons among groups were conducted using Tukey’s honest significant difference test. A *p*<0.05 was considered statistically significant. All data are expressed as means ± standard deviations (SD).

## Results

Body weight

**Figure 1:**
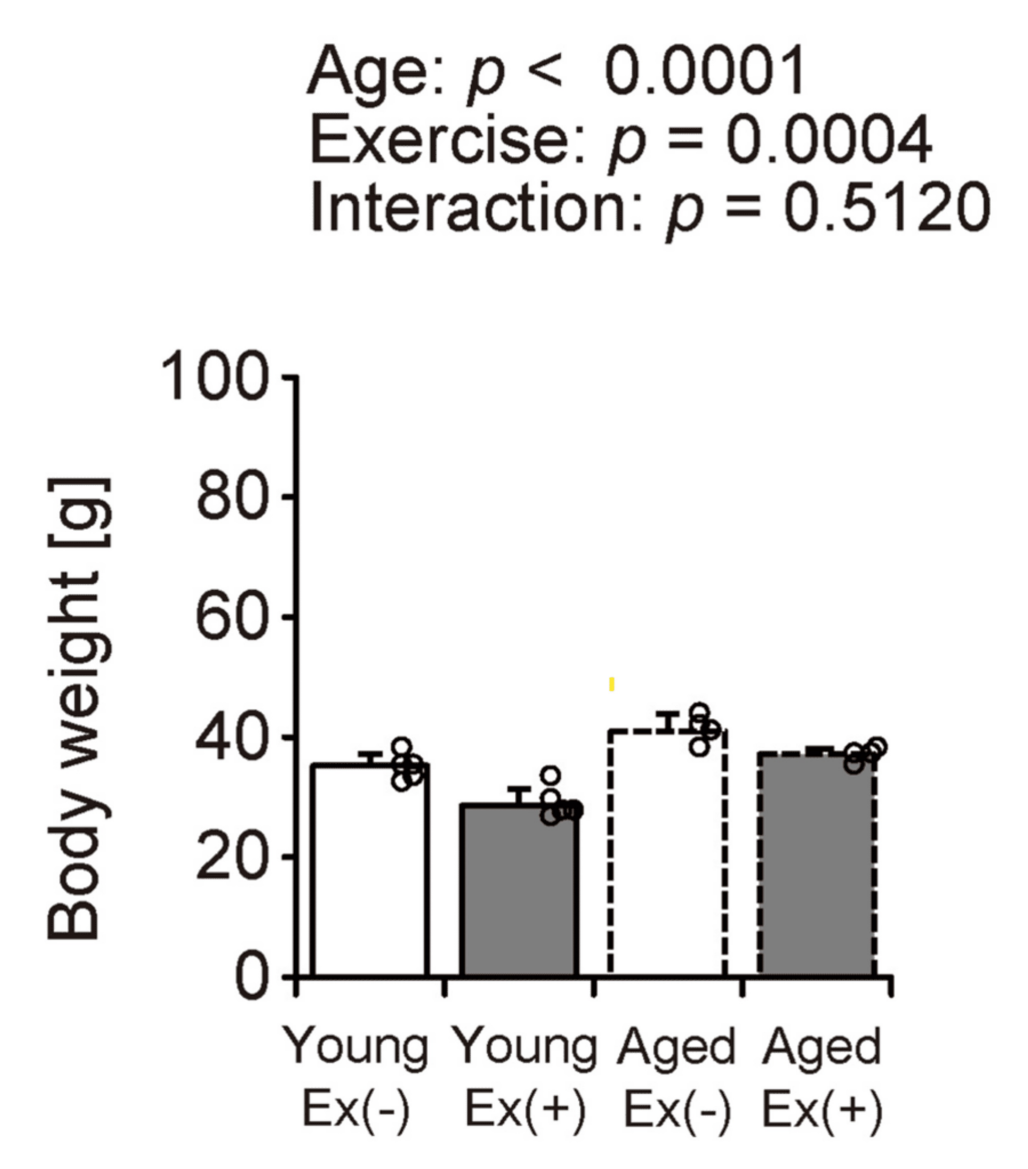
Body weight Values are presented as means ± standard deviations, with four to five animals per group. A two-way analysis of variance was conducted to assess the main effects of age and exercise, as well as their interaction. Data points are represented individually as circles with a white interior and black lines Ex: exercise

Body weight measurements were recorded to determine the effects of voluntary wheel running on body weight in young and aged mice maintained on an HFD (Figure 1). Body weight increases with age and decreases during voluntary exercise. These findings suggest that voluntary exercise may help mitigate the weight gain associated with an HFD.

Muscle weight

**Figure 2:**
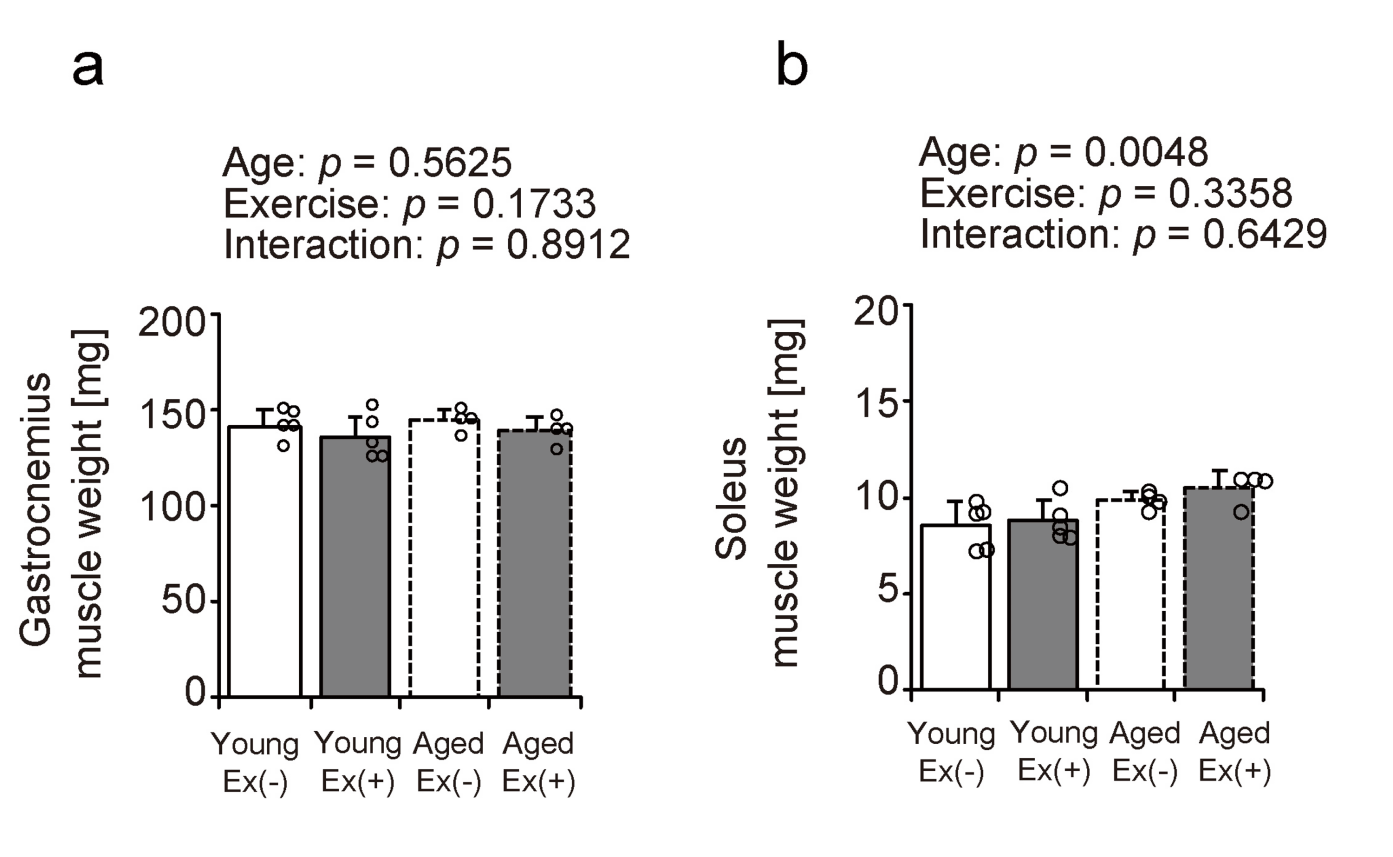
Muscle weight Values are presented as means ± standard deviations, with four to five animals per group. A two-way analysis of variance was conducted to assess the main effects of age and exercise, as well as their interaction (a, b). Data points are represented individually as circles with a white interior and black lines Ex: exercise

The weights of the gastrocnemius and soleus muscles were measured to assess the effects of aging and exercise on these muscles under HFD conditions (Figures 2a, 2b). No significant effects of aging or exercise were observed on the gastrocnemius muscle weight (Figure 2a). Soleus muscle weight tended to increase with age. No significant effects of exercise or age-exercise interactions were observed in the soleus muscle (Figure 2b). These findings suggest that voluntary exercise may have minimal influence on muscle mass in the context of an HFD.

Fiber cross-sectional area

**Figure 3:**
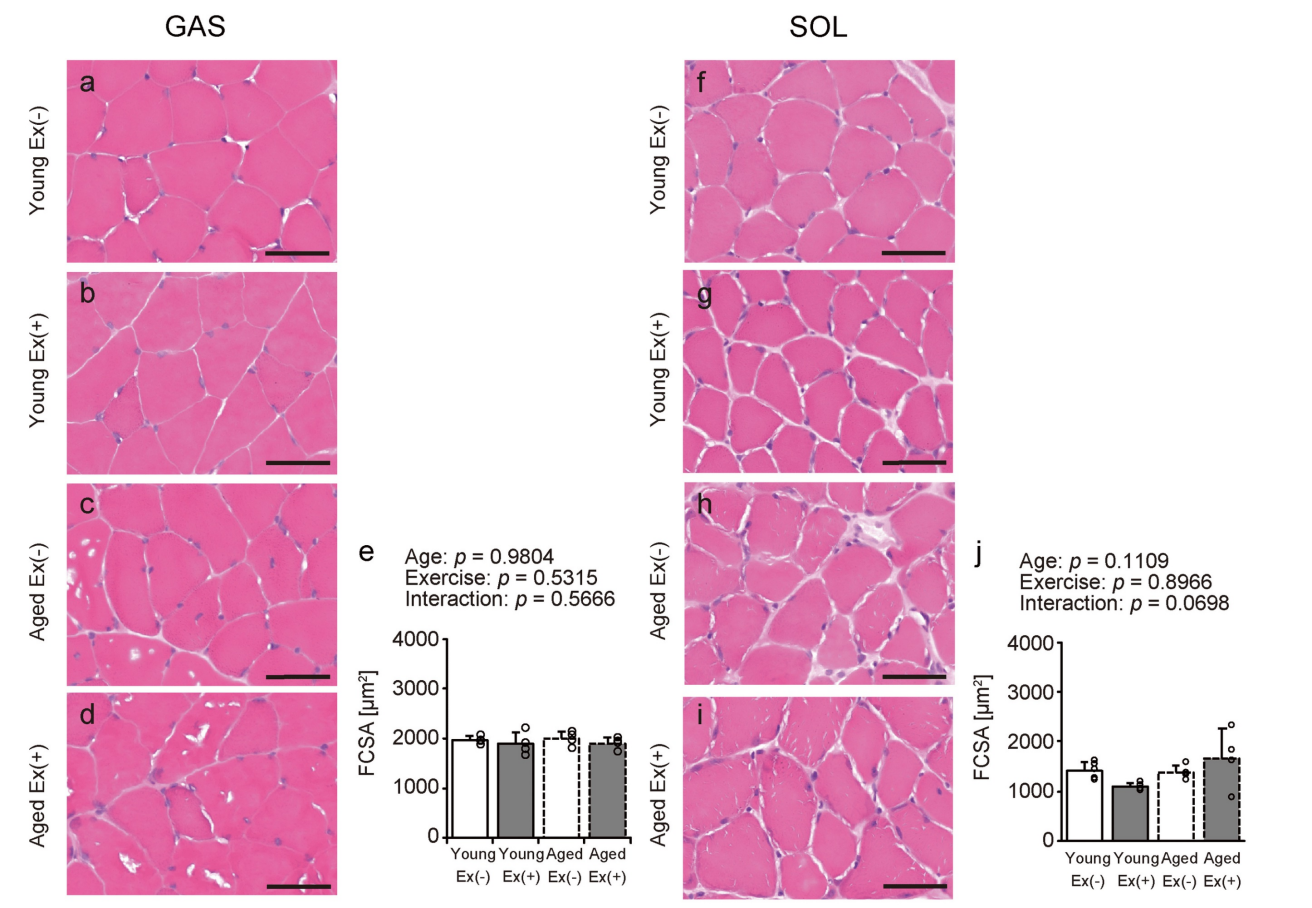
HE staining of gastrocnemius and soleus muscles. Representative transverse sections were stained with HE (a-d and f-i). Scale bar = 50 µm. The FCSA was quantified from the images (e, j). A two-way analysis of variance was conducted to assess the main effects of age and exercise, as well as their interaction (e, j). Values are presented as means ± standard deviations, with four animals per group. Data points are represented individually as circles with a white interior and black lines Ex: exercise; FCSA: fiber cross-sectional area; HE: hematoxylin and eosin

Transverse sections were stained with HE to assess the effects of aging and exercise on muscle fiber size under HFD conditions (Figures 3a-3d, 3f-3i), and the FCSA was quantified (Figures 3e, 3j). No significant effects of age and exercise were found on the FCSA in either muscle (Figures 3e, 3j). These findings suggest that the muscle fiber size in the gastrocnemius and soleus is not substantially affected by voluntary exercise under HFD conditions.

Succinate dehydrogenase staining

**Figure 4:**
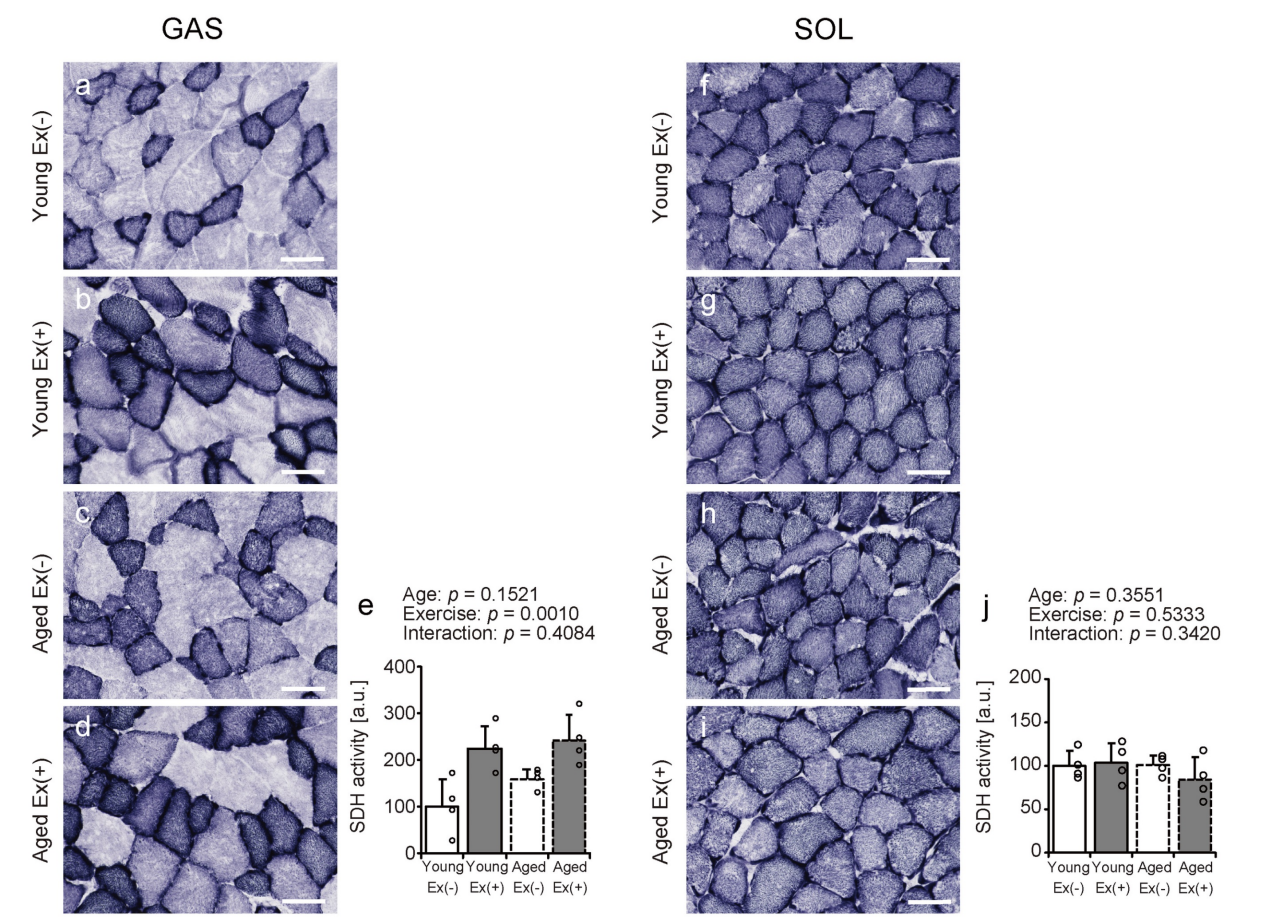
SDH staining of gastrocnemius and soleus muscles Representative transverse sections stained for SDH activity (a-d and f-i). Scale bar = 50 µm. SDH staining intensity was quantified (e, j). Values are presented as means ± standard deviations, with four animals per group. A two-way analysis of variance was conducted to assess the main effects of age and exercise, as well as their interaction (e, j). Data points are represented individually as circles with a white interior and black lines Ex: exercise; SDH: succinate dehydrogenase

To investigate the effects of age and voluntary exercise on mitochondrial metabolic activity in skeletal muscles under HFD conditions, SDH staining was applied (Figures 4a-4d, 4f-4i). Voluntary exercise tended to increase the SDH activity in the gastrocnemius muscle in both age groups (Figure 4e). No significant differences in age, exercise, or their interactions were detected in the soleus muscles (Figure 4j). These findings suggest that voluntary exercise may enhance mitochondrial metabolic activity in the gastrocnemius muscle of young and aged mice fed an HFD, while such a response may be scant in the soleus muscle.

Oil Red O staining

**Figure 5:**
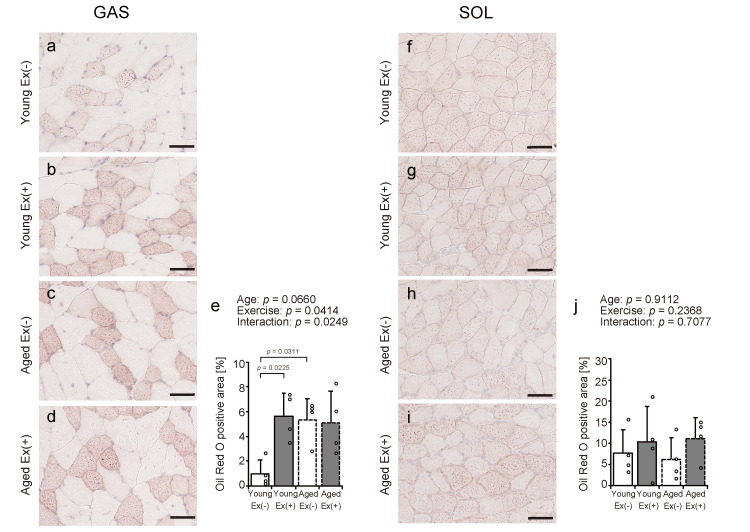
Oil Red O staining of gastrocnemius and soleus muscles Representative transverse sections were stained with Oil Red O (a-d and f-i). Scale bar = 50 µm. Stained areas were measured using images as Oil Red O-positive areas (e, j). Values are presented as means ± standard deviations, with four animals per group. A two-way analysis of variance was conducted to assess the main effects of age and exercise, as well as their interaction (e, j). A significant statistical interaction between age and exercise was indicated by Oil Red O-positive areas, followed by Tukey’s HSD test for post hoc multiple comparisons (e). Statistical significance was defined as *p* < 0.05. Data points are represented individually as circles with a white interior and black lines Ex: exercise

To investigate the effects of aging and exercise on intramuscular fat accumulation under HFD conditions, Oil Red O staining was used to visualize lipid droplets in the gastrocnemius and soleus muscles; the stained areas were quantified (Figures 5a-5j). Voluntary exercise led to a significant increase in Oil Red O-positive areas within the gastrocnemius muscle of young mice. In contrast, no such changes were observed in aged mice (Figure 5e). Aged sedentary mice showed higher fat accumulation than young sedentary mice (Figure 5e). No statistically significant variations were detected in the soleus muscle among groups that differed by age or exercise conditions (Figure 5j). These findings suggest that voluntary exercise under an HFD promotes gastrocnemius muscles intramuscular fat accumulation in young, but not aged, and that aging is associated with increased fat deposition regardless of exercise habits.

## Discussion

The present study investigated the effects of voluntary wheel running on skeletal muscle metabolic function and lipid infiltration in young and aged mice on an HFD. The main findings were as follows: (1) body weight increased with age and decreased with voluntary exercise; (2) voluntary exercise did not significantly alter skeletal muscle weight and FCSA in either age group; (3) voluntary wheel running enhanced SDH enzymatic activity in the gastrocnemius muscle in both age groups, whereas changes in the soleus muscle were minimal; and (4) exercise led to an increase in the intramuscular lipid content in the gastrocnemius muscle of young mice, but no such effect was observed in aged mice. These findings suggest that although voluntary exercise exhibits aerobic characteristics and contributes to weight reduction, intramuscular fat is dependent on muscle type and age during an HFD feeding.

Our study demonstrates that voluntary exercise under an HFD effectively reduces body weight in both young and aged mice. While human studies have shown that exercise alone may have limited effects on weight loss [19], previous research in mice suggested that exercise can increase energy expenditure, which may be compensated for by reduced non-exercise physical activity, potentially explaining the modest changes in body weight [20]. However, another study reported weight loss in young mice subjected to voluntary exercise while on an HFD [21]. These findings endorse the conclusion that voluntary exercise promotes weight loss in both young and aged mice fed an HFD. Second, voluntary exercise under an HFD did not significantly affect the weight or FCSA of the gastrocnemius and soleus muscles in either age group. While synergistic ablation is known to induce significant skeletal muscle hypertrophy through mechanical overload, aerobic exercises such as treadmill running have generally not been associated with hypertrophic effects or increased muscle mass [22]. These findings suggest that the intensity of voluntary exercise used in this study was insufficient to induce muscle hypertrophy.

Significantly, voluntary exercise under an HFD increased SDH activity across age groups in the gastrocnemius muscle; however, these changes were limited to the soleus muscle. SDH is a key enzyme involved in the tricarboxylic acid cycle that plays a critical role in mitochondrial oxidative metabolism [23]. SDH-knockout mice have been reported to exhibit reduced mitochondrial oxygen consumption, muscle fiber contractility, and exercise endurance [24]. Aerobic exercise increases SDH activity in the muscles and accelerates energy metabolism [25]. These findings suggest that spontaneous exercise may increase the mitochondrial metabolic activity of gastrocnemius muscles in both young and aged mice fed an HFD. The baseline SDH activity was greater in slow-twitch fibers than in their fast-twitch counterparts [26]. Therefore, mitochondrial metabolic activity may be accelerated by aerobic exercise stimulation in the gastrocnemius muscle, which is predominantly composed of fast-twitch fibers. In contrast, the soleus muscle, characterized by a high proportion of slow-twitch fibers, was originally high in mitochondrial activity and may have lacked the additional effects of voluntary exercise.

In this study, voluntary exercise under an HFD expanded the Oil Red O staining area in the young gastrocnemius muscle. Still, such changes were minimal in the gastrocnemius muscle of aged mice. The soleus muscle is poorly affected by voluntary exercise in both young and aged mice. Oil Red O staining was used to visualize intramuscular fat [27]. It has been reported that a high-fat, high-sucrose diet leads to enhanced Oil Red O staining, reflecting greater intramuscular fat deposition [28]. A previous study investigating the effects of exercise on intramuscular fat has reported that skeletal muscles from endurance-trained athletes contain more intramuscular fat, which may be used for energy production through fat oxidation [29]. Thus, voluntary exercise in young mice may stimulate intramuscular fat storage to increase energy expenditure. In contrast, the lack of this response in aged mice may reflect age-related impairments in lipid metabolism and storage mechanisms in skeletal muscle. Another previous study in mice reported that aging and an HFD intake led to the accumulation of intramuscular fat [30]. Based on these findings, it is possible that aged mice fed an HFD were more likely to have increased intramuscular fat mass, and that the effect of voluntary exercise was limited.

Limitations

This study has several limitations. Primarily, the exclusive focus was on the histological evaluation of skeletal muscle. Molecular-level analyses can provide deeper insights into the underlying mechanisms related to changes in muscle metabolic activity. Second, this study specifically compared the influence of voluntary exercise under HFD conditions between young and aged mice; however, it did not evaluate the impact of alternative dietary compositions or exercise modalities. For instance, an experimental system that includes a regular diet, a high-sugar diet, and forced exercise may provide more comprehensive insights into how dietary and physical activity variables influence skeletal muscles, especially in the context of aging. Third, with the installation of running wheels in the cages of the exercise group, all groups were housed individually in cages used for the rats. If cage size affects the amount of activity in mice, it may also affect the degree of obesity due to an HFD. Further research is needed to determine whether cage size affects skeletal muscle function in aging individuals. The amount of activity a cage provides may have an impact on skeletal muscles. Fourth, the aged mice used in this study were in a condition before the onset of significant age-related muscle mass loss compared with young mice. Future experiments with aged mice, using monthly mice in a state of apparent sarcopenia, may provide a better understanding of the effects of spontaneous exercise on aging. Fifth, the muscle samples targeted in this study were lower extremity muscles, and the effects of upper extremity and trunk muscles were not confirmed. Evaluation of systemic muscles has the potential to provide insight into exercise effects by muscle localization and type. Finally, the number of subjects was limited in consideration of the 3R principle and the difficulty of obtaining aged animals. Therefore, caution should be exercised when generalizing the results obtained.

## Conclusions

The present study aimed to elucidate the differential effects of voluntary exercise on skeletal muscle morphology in young and aged mice on an HFD. The study findings indicate that voluntary exercise increases mitochondrial activity in the gastrocnemius muscles in both age groups. However, it had minimal effects on the muscle weight and FCSA in both young and aged mice. Exercise-induced changes in intramuscular fat content varied depending on muscle type and age. Further analyses of the molecular mechanisms by which voluntary physical activity modulates intramuscular lipid accumulation and metabolic function in the context of HFD are warranted.
